# Investigating the Pea Virome in Germany—Old Friends and New Players in the Field(s)

**DOI:** 10.3389/fmicb.2020.583242

**Published:** 2020-11-13

**Authors:** Yahya Z. A. Gaafar, Kerstin Herz, Jonas Hartrick, John Fletcher, Arnaud G. Blouin, Robin MacDiarmid, Heiko Ziebell

**Affiliations:** ^1^Julius Kühn Institute, Institute for Epidemiology and Pathogen Diagnostics, Braunschweig, Germany; ^2^The New Zealand Institute for Plant and Food Research Limited, Auckland, New Zealand; ^3^School of Biological Sciences, The University of Auckland, Auckland, New Zealand

**Keywords:** *Pisum sativum*, high throughput sequencing, emaravirus, aphid transmitted viruses, PEMV, PNYDV, TuYV

## Abstract

Peas are an important legume for human and animal consumption and are also being used as green manure or intermediate crops to sustain and improve soil condition. Pea production faces constraints from fungal, bacterial, and viral diseases. We investigated the virome of German pea crops over the course of three successive seasons in different regions of pea production to gain an overview of the existing viruses. Pools from 540 plants, randomly selected from symptomatic and asymptomatic peas, and non-crop plants surrounding the pea fields were used for ribosomal RNA-depleted total RNA extraction followed by high-throughput sequencing (HTS) and RT-PCR confirmation. Thirty-five different viruses were detected in addition to nine associated nucleic acids. From these viruses, 25 are classified as either new viruses, novel strains or viruses that have not been reported previously from Germany. Pea enation mosaic virus 1 and 2 were the most prevalent viruses detected in the pea crops, followed by pea necrotic yellow dwarf virus (PNYDV) and turnip yellows virus which was also found also in the surrounding non-legume weeds. Moreover, a new emaravirus was detected in symptomatic peas in one region for two successive seasons. Most of the identified viruses are known to be aphid transmissible. The results revealed a high virodiversity in the German pea fields that poses new challenges to diagnosticians, researchers, risk assessors and policy makers, as the impact of the new findings are currently unknown.

## Graphical Abstract

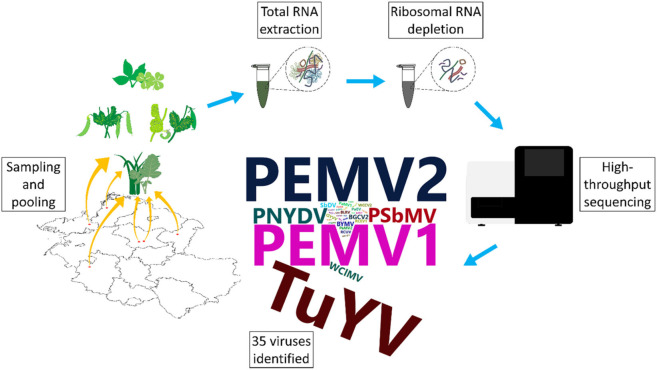

## Introduction

Green peas (*Pisum sativum* L.) are popular vegetables in Germany. The production of green peas increased from 4,444 ha in 2010 to 5,488 ha in 2018 (Behr, 2015, 2019). In addition, owing to the “Protein Strategy” of the Federal Government of Germany, the production areas of protein peas used as animal fodder, green manure or as intermittent crops, increased from 57,200 ha in 2010 to 70,700 ha in 2018 (BMEL, 2019). However, depending on the intended use of the crop, pea production in Germany is highly regionalized. The main green pea production areas are in Saxony, because of the nearby frozen foods processing facilities. Seed production of peas is predominantly carried out in Saxony-Anhalt. By contrast, green pea production for the fresh market or protein pea production for animal fodder/green manure is scattered around the country and often associated with small-scale or organic farming.

Pea plants are known to be hosts to several viruses from different families, e.g., *Luteoviridae*, *Nanoviridae*, and *Potyviridae*. These viruses often occur in mixed infections causing a range of symptoms such as chlorosis, dwarfing, mottling, vein clearing, enations, and necrosis (Musil, 1966; Bos et al., 1988; Kraft, 2008; Gaafar et al., 2016; Gaafar and Ziebell, 2019b). Because of the recent detection of novel pea-infecting viruses, such as pea necrotic yellow dwarf virus (PNYDV), across Germany and within neighboring countries (Grigoras et al., 2010a; Gaafar et al., 2016, 2017, 2018) we were interested to know whether there are more unknown and/or undetected viruses present in this crop in Germany.

Currently, virus diagnostics generally depend on observational, serological or molecular methods that are based on prior knowledge of the target virus. These methods do not address the potential presence of other viruses that may contribute to the etiology of a disease. In recent years, high-throughput sequencing (HTS) has enabled the identification of many new viruses from domesticated and wild plants (Roossinck et al., 2015; Gaafar et al., 2019c, d). HTS allows sequencing of all the genetic material in a given sample. Therefore there is no need for prior knowledge of the infectious agent (Adams et al., 2009; Roossinck et al., 2015; Maree et al., 2018). Improvements of HTS technologies and bioinformatic tools have helped to identify the virus community or viromes of several crops (Coetzee et al., 2010; Czotter et al., 2018; Jo et al., 2018). The generated data reveal a description of plant virus biodiversity, allow the discovery of new viruses and viroids, identify genomic variants of the viral species and aid the development of specific and sensitive diagnostics (Coetzee et al., 2010; Li et al., 2012; Gaafar et al., 2019a, b). However, the vast number of new viruses identified by HTS results in challenges for diagnosticians, pest risk assessors and policymakers. The actual risks to crop plants and alternative hosts of these new viruses need to be evaluated and explored (MacDiarmid et al., 2013; Massart et al., 2017; Rott et al., 2017; Maree et al., 2018). We believe the metagenomics data revealed by HTS, along with biological studies will help us better understand viral ecology, viral evolution and disease epidemiology, particularly in economically important crops. Nevertheless, HTS studies impose a huge challenge on risk assessors and policy makers as the “real” importance of the findings (Does the presence of a novel virus cause a problem to the crop plant it was found in? What is the “real” distribution/quantity of a potential threatening virus in the whole crop?) cannot be assessed from sequence data alone.

In this study, using HTS we explored the spatio-temporal changes of the pea virome in six German regions with different production foci, including frozen produce, protein pea seed production, seed production and breeding over a period of three seasons. We also investigated alternative virus reservoirs of legume and non-legume weeds associated with these production sites. To our knowledge, this is the first metagenomics study of crop plants incorporating spatio-temporal developments.

## Materials and Methods

### Sampling

Six regions in Germany were chosen for sampling and included Salzlandkreis-1: pea seed production, Salzlandkreis-2: trial site and heritage pea collection, Münster: trial site pea breeding, Kreis Stormarn: protein pea production, Landkreis Rostock: trial site for green manure mixtures, and Landkreis Meißen: green pea frozen production for human consumption.

**FIGURE 1:**
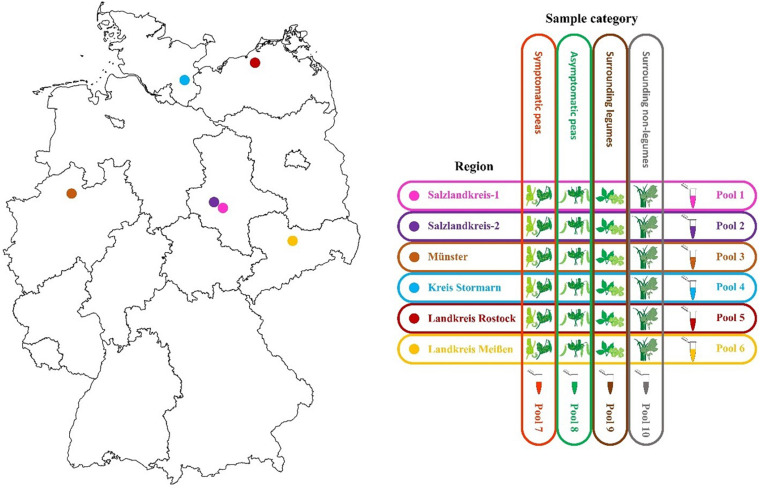
Map of the six different pea crop sampling regions in Germany and the sample pooling strategy used for each of three successive seasons 2016, 2017, and 2018.

A typical production field was chosen and based on long-term experience, samples were selected randomly over three successive seasons (2016, 2017, and 2018 between June and July; Figure 1). From each field, 10 obviously symptomatic pea plants (SP) and 10 asymptomatic (no obvious symptoms) pea plants (aSP) were selected randomly; in addition to the pea plants, five surrounding legume plants (sL), and five non-legume plants (snL), were also collected at random. The metadata were recorded, including location, sample, season category, plant host, symptoms, and the average seasonal temperature for that region (Supplementary Table S1). Any deviations from the sampling strategy (i.e., in cases where no non-symptomatic peas or no surrounding legume plants could be detected) were noted (Supplementary Table S1).

Ten sample pools were prepared each season, i.e., six separate pools containing material from each of the six regions (30 plants each) and four separate pools comprising material from all SP (60 plants), aSP (60 plants), sL (30 plants), or snL (30 plants), respectively, from all regions (Figure 1). Fresh tissue (100 mg) from each plant was pooled. The samples were mixed and ground using a mortar and pestle under liquid nitrogen then collected into 50 mL Falcon tubes. The pools were stored at −20°C until RNA extraction. Remaining sample tissue was also stored at −20°C for further analysis.

### RNA Extraction and High Throughput Sequencing

Total RNA was extracted using innuPREP Plant RNA Kit (Analytik Jena) from three subsamples of 100 mg from each pool to ensure sufficient total RNA yield for further processing thus allowing detection of low titre viruses. The extracted subsets were mixed and used for ribodepletion. Ribosomal RNA (rRNA) was depleted using a RiboMinus^TM^ Plant Kit for RNA-Seq (Invitrogen). cDNA was synthesized using ProtoScript II Reverse Transcriptase (NEB) and random octanucleotide primers (8N), followed by second strand synthesis using a NEBNext Ultra II Non-Directional RNA Second Strand Synthesis Module (NEB). The libraries were prepared from the double-stranded cDNA using Nextera XT Library Prep Kit (Illumina). The HT sequencing was performed on an Illumina MiSeq platform (301 × 2).

### Bioinformatic Analysis

Bioinformatic analysis was performed using Geneious Prime software (version 2019.1.1). The reads were quality trimmed and normalized. *De novo* assembly was performed and the resulting contigs were compared against a local database of viruses and viroids sequences downloaded from NCBI using Blastn and Blastx (*E*-value = 1e-5) (downloaded 13 August 2018). The generated consensus sequences were based on the highest quality threshold. Primers for virus validation were designed using a modified version of Primer3 (2.3.7) tool in Geneious Prime (Untergasser et al., 2012). Pairwise alignments were performed using Clustal W tool (v 2.1) in Geneious (Larkin et al., 2007). Neighbor joining phylogenetic trees were constructed using MEGA X software (Kumar et al., 2018). The phylogenetic relationships were established according to the species demarcation criteria set by the International Committee on Taxonomy of Viruses (ICTV), using the nucleotide sequences or the amino acid sequences of the capsid protein (CP) or the RNA dependent RNA polymerase (RdRP) for the respective families. The isolates were named by region number and season, e.g., R1_16 stands for region one and the season 2016. The assembled virus sequences can be accessed in GenBank under accession nos. MN314973, MN399680–MN399748, MN412725–MN412751, and MN497793–MN497846.

### RT-PCR Confirmations

For virus identity confirmation, total RNA was re-extracted from each pool as described above, followed by RT-PCR with the primers listed in Supplementary Table S2 and using the OneTaq One-Step RT-PCR Kit (NEB). The products were purified using Zymoclean Gel DNA Recovery Kit (Zymo Research) and Sanger sequenced using both RT-PCR primers.

### Statistical Analysis

Statistical analysis was performed using scripts written on R (version 3.5.3) (R Core Team, 2019). The virus incidence was calculated for each virus and associated nucleic acids, where a score of 1 is the lowest, for detected once, and a score of 78 is the highest, when a virus was detected in each sampling category of all regions over all seasons.

## Results

### Metadata and HTS Raw Data

Symptoms observed on peas in the “symptomatic” pea pool included plant dwarfing, stunting of top leaves, translucent leaf spotting and leaf enations, leaf yellowing, leaf mosaic and mottling, leaf rolling and pod distortion (Supplementary Table S1). In 2016 leguminous weeds appeared absent in fields surrounding Salzlandkreis-1 and no asymptomatic peas were found on the site in Salzlandkreis-2. Similarly, leguminous weeds also appeared absent in Landkreis Meißen in 2018. Consequently, these pools were omitted from the analyses. Raw data generated from the HTS MiSeq platform are summarized in Supplementary Table S3.

### The Viruses Detected in German Pea Fields

**FIGURE 2:**
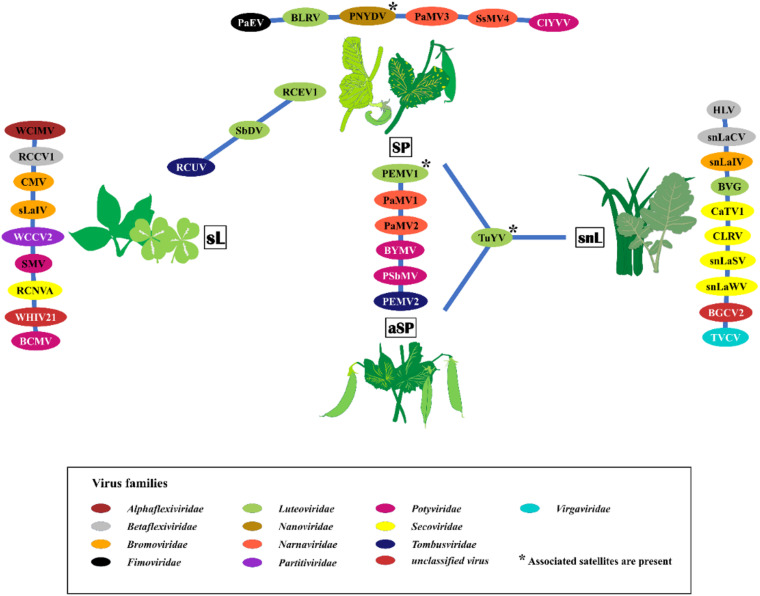
The virus species found in the four sampling categories symptomatic peas (SP), asymptomatic peas (aSP), surrounding legumes (sL), and surrounding non-legumes (snL). Samples were collected in six pea-growing regions (Salzlandkreis-1, Salzlandkreis-2, Münster, Kreis Stormarn, Landkreis Rostock, and Landkreis Meißen) in Germany over three successive seasons 2016, 2017, and 2018. A network illustration of virus species of each category and shared viruses among them. Background colors of virus acronyms correspond to the background colors of the respective virus family. The virus names are: BVG, barley virus G; BCMV, bean common mosaic virus; BLRV, bean leafroll virus; BYMV, bean yellow mosaic virus; BGCV2, black grass cryptic virus 2; CaTV1, carrot torradovirus 1; CLRV, cherry leaf roll virus; ClYVV, clover yellow vein virus; CMV, cucumber mosaic virus; HLV, Heracleum latent virus; PaMV1, pea associated mitovirus 1; PaMV2, pea associated mitovirus 2; PaMV3, pea associated mitovirus 3; PaEV, pea associated emaravirus; PEMV1, pea enation mosaic virus 1; PEMV2, pea enation mosaic virus 2; PNYDV, pea necrotic yellow dwarf virus; PSbMV, pea seed-borne mosaic virus; RCCV1, red clover carlavirus 1; RCEV1, red clover enamovirus 1; RCNVA, red clover nepovirus a; RCUV, red clover umbravirus; SsMV4, Sclerotinia sclerotiorum mitovirus 4; sLaIV, sL associated ilarvirus; snLaCV, snL associated chordovirus; snLaIV, snL associated ilarvirus; snLaSV, snL associated secoviridae; snLaWV, snL associated waikavirus; SbDV, soybean dwarf virus; SMV, soybean mosaic virus; TVCV, turnip vein-clearing virus; TuYV, turnip yellows virus; WCCV2, white clover cryptic virus 2; WClMV, white clover mosaic virus; WHIV21, Wuhan insect virus 21.

Thirty-five viruses were detected by HTS and further confirmed by RT-PCR in the sample pools over the three seasons. In addition, several other viral reads were detected that could not be confirmed by RT-PCR or that were obvious contaminations from various samples that were placed onto the same sequencing run (Supplementary Table S6). The detected viruses represented 14 different families and several unassigned viruses (Figure 2). The family *Luteoviridae* was represented by seven species members, followed by *Secoviridae* with six members, and *Potyviridae* with five members. Over the survey, the widest virus diversity was recorded in the SP pools, with sixteen different virus species present. The sL pool contained 12 different virus species, the snL pools included 11 virus species, and the aSP recorded seven virus species (Figure 2).

The identified viruses, the regions in which they were detected, and the pools from which they were discovered, are listed in Supplementary Table S4. The incidence of the detected viruses was calculated for each virus (Supplementary Table S4). Turnip yellows virus (TuYV) was the most abundant virus in this study with a score of 33, followed by pea enation mosaic virus 2 (PEMV2, score 31), pea enation mosaic virus 1 (PEMV1, score 29). Pea necrotic yellow dwarf virus (PNYDV) had a score of 11 followed by pea seed-borne mosaic virus (PSbMV) with a score of 10.

**FIGURE 3:**
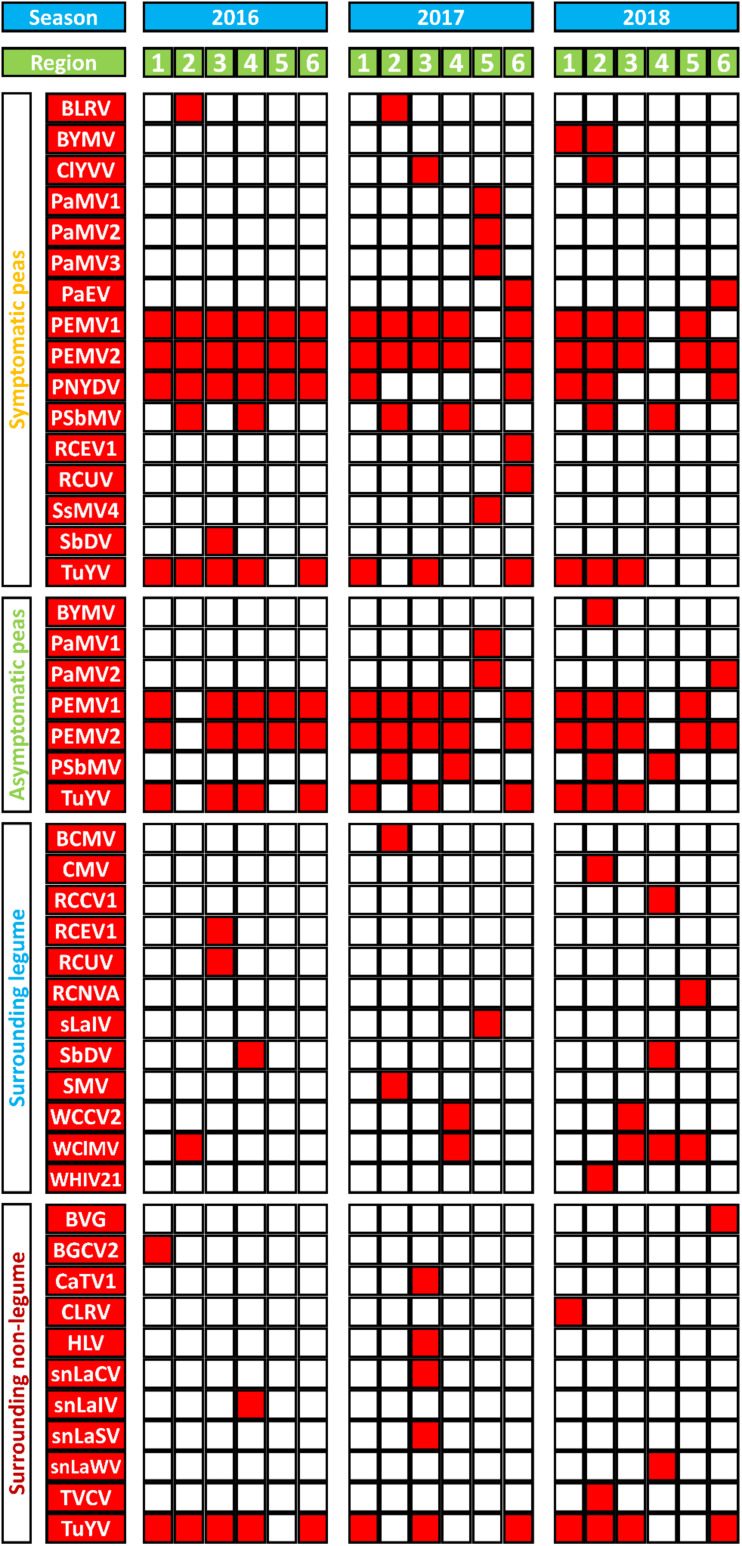
A summary of virus species (in red) detected in and around sampled pea crops from six different German pea-growing regions (Salzlandkreis-1 [1], Salzlandkreis-2 [2], Münster [3], Kreis Stormarn [4], Landkreis Rostock [5], and Landkreis Meißen [6]) over three seasons 2016, 2017, and 2018. Virus acronyms, virus names, and their associated nucleic acids: BVG, barley virus G; BCMV, bean common mosaic virus; BLRV, bean leafroll virus; BYMV, bean yellow mosaic virus; BGCV2, black grass cryptic virus 2; CaTV1, carrot torradovirus 1; CLRV, cherry leaf roll virus; ClYVV, clover yellow vein virus; CMV, cucumber mosaic virus; HLV, Heracleum latent virus; PaMV1, pea associated mitovirus 1; PaMV2, pea associated mitovirus 2; PaMV3, pea associated mitovirus 3; PaEV, pea associated emaravirus; PEMV1, pea enation mosaic virus 1; PEMV2, pea enation mosaic virus 2; PEMVsatRNA, pea enation mosaic virus satellite RNA; PNYDVaSat1, pea necrotic yellow dwarf alphasatellite 1; PNYDVaSat3, pea necrotic yellow dwarf alphasatellite 3; PNYDVaSat4, pea necrotic yellow dwarf alphasatellite 4; PNYDVaSat5, pea necrotic yellow dwarf alphasatellite 5; PNYDVaSat6, pea necrotic yellow dwarf alphasatellite 6; PNYDVaSat7, pea necrotic yellow dwarf alphasatellite 7; PNYDV, pea necrotic yellow dwarf virus; PSbMV, pea seed-borne mosaic virus; RCCV1, red clover carlavirus 1; RCEV1, red clover enamovirus 1; RCNVA, red clover nepovirus a; RCUV, red clover umbravirus; SsMV4, Sclerotinia sclerotiorum mitovirus 4; sLaIV, sL associated ilarvirus; snLaCV, snL associated chordovirus; snLaIV, snL associated ilarvirus; snLaSV, snL associated secoviridae; snLaWV, snL associated waikavirus; SbDV, soybean dwarf virus; SMV, soybean mosaic virus; TVCV, turnip vein-clearing virus; TuYV, turnip yellows virus; TuYVaRNA, turnip yellows virus associated RNA; TuYVaRNA2, turnip yellows virus associated RNA 2; WCCV2, white clover cryptic virus 2; WClMV, white clover mosaic virus; WHIV21, Wuhan insect virus 21.

Figure 3 summarizes virus occurrence in peas (SP and aSP) and the surrounding non-crop plants (sL and snL) in each region over the three seasons. If looking at the pea pools only, PEMV2 was the most abundant virus with the score of 31 followed by PEMV1 (score 29) because they were not present in sL nor snL pools. With a score of 21, TuYV is the third most abundant virus in the pea pools.

In the sL, white clover mosaic virus (WClMV) was the most abundant virus, with a score of 5, followed by white clover cryptic virus 2 (WCCV2) and soybean dwarf virus (SbDV) with a score of 2 (Figure 3). All the other listed viruses were detected only once. In the snL samples, TuYV was the most abundant virus with a score of 12, while all the other viruses were detected only once.

### The Most Abundant Viruses Detected in German Peas

TuYV was the most abundant virus from the study and was detected not only in pea (SP and aSP) but also in snL pools over the three seasons. Nucleotide identity (nt%) showed 95–99.2% to published TuYV sequences (Supplementary Table S5). Two associated RNAs, TuYV-associated RNA (TuYVaRNA) and a novel associated RNA (TuYVaRNA2), were also detected. TuYVaRNA2 isolates were closely related to cucurbit aphid borne virus-associated RNA (CABYVaRNA; KM486094, from the United States), with 89.3 to 95.1% nt identity (Supplementary Table S5).

Over the observation period, PEMV1 was detected almost all the time in all regions in SP and aSP pools with the exceptions of region 5 in 2017 and region 4 in 2018, where neither PEMV1 nor PEMV2 could be detected. The nucleotide (nt) sequences of PEMV1 isolates showed highest identities (96.1–98.9%) to MK948533 (Supplementary Table S4). In general, PEMV2 was always associated with PEMV1, with the exception of region 6 in 2018 where only PEMV2 was found. The nt sequences of PEMV2 showed 93.5–94.4% nt identity to MK948534 (Supplementary Table S4). In addition, we also detected a satellite RNA (PEMVSatRNA). Neighbor joining trees (NJ) based on the readthrough RdRp aa sequences of the isolates of the luteovirids and tombusvirids detected in German pea fields over three seasons are shown in Supplementary Figures S1, S2.

PNYDV was detected in SP pools with nt identities between 99.2 and 99.9% with PNYDV sequences available on NCBI (Supplementary Table S5). Six PNYDV alphasatellites (PNYDVαSat) including alphasatellites 1 and 3, and four new alphasatellites, tentatively named PNYDV-associated alphasatellites 4, 5, 6, and 7, were also detected. The isolates of PNYDVαSat4, PNYDVαSat5, PNYDVαSat6 and PNYDVαSat7 were 1,030, 991, 1,037, and 1,015 nt in length, respectively. PNYDVαSat4 showed closest nt identity to faba bean necrotic yellows virus C7 alphasatellite (FBNYC7αSat; AJ005964) from Egypt, with 88.5 and 88.6% relationship. Phylogenetic trees based on the alignments of the nt sequences and the aa sequences of the encoded proteins are shown in Supplementary Figures S3A,B.

PSbMV was detected only in two regions, Salzlandkreis-2 and Kreis Stormarn, but was present over all three seasons in SP and aSP pools. PSbMV isolates had 96.2–99.7% nt identity to D10930, a Danish isolate. Neighbor joining tree based on the polyprotein aa sequences of the potyvirid isolates detected in German pea fields over three seasons are shown in Supplementary Figure S4.

#### New Viruses and Virus Strains

##### A New Emaravirus in German Peas (Fimoviridae): Pea Associated Emaravirus (PaEV)

**FIGURE 4:**
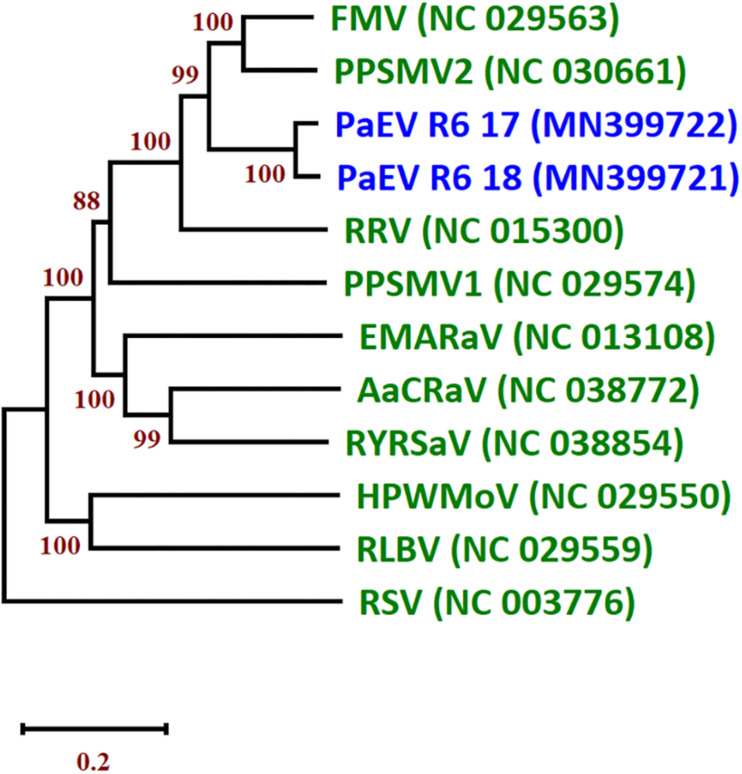
Neighbor joining tree (NJ) of the nucleocapsid protein (NP) of the two isolates of pea associated emaravirus (PaEV) detected in Germany and representative emaraviruses. The phylogenetic trees are based on alignments of the amino acid sequences. The sequences were aligned with Clustal W and NJ trees constructed with MEGA X. The percentage of the bootstrap values above 50% (1,000 replications) are shown at the nodes. Virus acronyms and names are as follows: AcCRaV, Actinidia chlorotic ringspot-associated emaravirus; EMARaV, European mountain ash ringspot-associated emaravirus; FMV, fig mosaic emaravirus; HPWMEV, high plains wheat mosaic emaravirus; PaEV, pea associated emaravirus; PPSMV1, pigeonpea sterility mosaic emaravirus 1; PPSMV2, pigeonpea sterility mosaic emaravirus 2; RLBV, raspberry leaf blotch emaravirus; RRV, rose rosette emaravirus; RSV, rice stripe virus (*Tenuivirus; Phenuiviridae*) and RYRSaV, redbud yellow ringspot-associated emaravirus. The GenBank accession numbers are in brackets. The scale bar indicates the genetic distance.

A new emaravirus was identified in SP pools from of Landkreis Meißen in 2017 and 2018. The virus showed high similarity to other members of genus *Emaravirus* including fig mosaic virus (FMV), pigeonpea sterility mosaic virus 2 (PPSMV2) and rose rosette virus (RRV). Members of the genus *Emaravirus* (family *Fimoviridae*, order *Bunyavirales*) have segmented, linear, single-stranded, negative-sense RNA genomes (Elbeaino et al., 2018). Their genomes are composed of up to eight segments. Partial sequences of segments RNA1–6 were assembled from the two Meißen isolates. An NJ tree of the nucleocapsid protein (NP) amino acid (aa) sequences of the two isolates was constructed and grouped together in a clade with PPSMV2 and FMV (Figure 4). The aa sequence of the NP shared the highest identity with PPSMV2 with 71.7%. According to the species demarcation of ICTV, a difference of 25% in the aa sequence of the NP indicates a new species (Elbeaino et al., 2018). Therefore, this virus represents a new emaravirus species and is tentatively called pea associated emaravirus (PaEV). To identify the pea sample in which PaEV was present, total RNA was extracted from individual samples from the positive pool of 2018 and virus presence was confirmed by RT-PCR in two samples with chlorosis symptoms. These samples were also infected with PEMV2 and PNYDV as confirmed by RT-PCR. Supplementary Figure S5 shows the symptoms on sample R6-18-05 from Landkreis Meißen in 2018.

##### Pea Associated Mitoviruses

In Landkreis Rostock, four mitoviruses (family: *Narnaviridae*) were detected in the SP and aSP pools in 2017. An isolate of Sclerotinia sclerotiorum mitovirus 4 (SsMV4) was identified from the SP pool. This isolate shared 96.3% aa identity to SsMV4 from New Zealand (AGC24233). The three further mitoviruses identified are provisionally called pea associated mitovirus 1, 2 and 3 (PaMV1, PaMV2 and PaMV3). The PaMV1-CDS shared closest identity of 68.8% nt to Erysiphe necator mitovirus 3 (EnMV3; KY420040), identified from the grape powdery mildew fungus *Erysiphe necator* (Schwein.) [syn. *Uncinula necator* (Schw.)], recently described in the United States (Pandey et al., 2018). Based on the RdRp region, PaMV1 shares 65.1% aa identity with EnMV3. PaMV2-CDS shares 36% nt identity to Rhizoctonia solani mitovirus 6 (RsMV6; KP900915 from the United States). Based on the aa sequences, PaMV1 and RsMV6 share only 40.2% aa identity. PaMV3-CDS was closely related to Entomophthora muscae mitovirus 5 (EnmuMV5; MK682524), with 40% nt identity and 24.3% aa identity to the RdRp region.

##### Other Novel Virus Detections

Twenty-three viruses were detected from the sL and snL pools, and of these, five viruses appear to be novel. These new viruses were provisionally named according to the particular pool in which they were detected. The closest GenBank sequences of these viruses are presented in Supplementary Table S5. Two new ilarviruses were identified in the snL samples of Trenthorst in 2016; one appears closely related to asparagus virus 2, the other from the sL pool of Landkreis Rostock in 2018 appears to be closely related to ageratum latent virus. The putative CP aa of this snL ilarvirus shared closest identity to asparagus virus 2 (AV2; NC_011807) with 81.1% aa identity. The sL ilarvirus CP was closely related to ageratum latent virus (AgLV; NC_022129) with 60.3% aa identity. A phylogenetic tree based on the alignments of the RdRp aa sequences of the bromovirids is shown in Supplementary Figure S6.

In Münster 2017, a new putative member of the family *Secoviridae* with a close relationship to lettuce secovirus 1 was also identified in snL. The snLSV protease-polymerase region (Pro-Pol) shared closest aa identity to LSV1 with 67.3% while its CP region showed closest aa identity to LSV1 with 31.6%. Neighbor joining tree based on the Pro-Pol aa sequences of the secovirids detected in German pea fields over three seasons are shown in Supplementary Figure S7. We also detected a partial sequence of a chordovirus (subfamily: *Trivirinae*) in the snL of Münster 2017, sharing 74.4% identity with carrot chordovirus 1. Finally, in 2018, a partial waikavirus sequence was detected from the snL pool of Kreis Stormarn and appears to be related to bellflower vein chlorosis virus. A phylogenetic tree based on the alignments of the replicase aa sequences of the betaflexivirids is shown in Supplementary Figure S8.

In addition to the viruses described above, new virus strains were detected including red clover enamovirus 1 (RCEV1), red clover umbravirus (RCUV), red clover nepovirus A (RCNVA), red clover carlavirus 1 (RCCV1) and Wuhan insect virus 21 (WHIV21) were identified in the sL, and divergent isolates of cherry leaf roll virus (CLRV) and carrot torradovirus 1 (CaTV1) were found in the snL. Also, the complete coding sequence of an isolate of Heracleum latent virus (HLV) (genus: *Vitivirus*; subfamily: *Trivirinae*, family: *Betaflexiviridae*), was identified in snL of Münster 2017.

In Münster 2016, a new strain of red clover enamovirus 1 (RCEV1) was identified in the sL samples. Its RdRp aa sequence shows 87.1% identity to the Czech strain (MG596229). Another isolate was detected in Landkreis Meißen in 2017, with 87.6% nt aa identity to each other and 95.8% to the Czech strain. The viruses grouped together within the *Enamovirus* genus clade (Supplementary Figure S1). A new strain of RCUV, a novel umbravirus found in red clover from the Czech Republic (Koloniuk, pers. comm.), was detected in the sL in Münster 2016 and the SP in Landkreis Meißen 2017. The complete coding sequence (CDS) shared 87.1% nt identity, while the aa sequence of their RdRp shared to 91.6% aa identity to the Czech isolate (MG596234). Both virus strains are grouped together within the *Umbravirus* genus (Supplementary Figure S2).

A divergent strain of red clover carlavirus 1 (RCCV1) (genus: *Carlavirus*; subfamily: *Quinvirinae*; family: *Betaflexiviridae*), was identified in one location (Kreis Stormarn) in 2018 only. The partial RdRp sequence shared 85.3% aa identity with RCCV1 (MG596238 and MG596239) from the Czech Republic. The CDS of an isolate of Heracleum latent virus (HLV) (genus: *Vitivirus*; subfamily: *Trivirinae*, family: *Betaflexiviridae*), was identified the snL of Münster 2017. Based on the CP sequence, this isolate shared 90.9% identity to HTV (NC_039087) on the nt level and 96.4% identity based on the aa sequence. Moreover, the RdRp shared 58.4% nt identity to grapevine virus B (GVB; MF991949) and 58.9% aa identity.

In addition, a new strain of RCNVA (genus: *Nepovirus*; subfamily: *Comovirinae*; family: *Secoviridae*), was detected in Landkreis Rostock 2017. This new strain was identified with Pro-Pol aa identity of 96.5% to MG253828 and CP aa identity of 83.2% to MG253829. A divergent cherry leaf roll virus (CLRV) (genus: *Nepovirus*) was identified in Salzlandkreis-1 in 2018. The virus shared closest identity with CLRV isolates from New Zealand, where RNA1 has 82.4% nt identity to CLRV isolates KC937022 and RNA2 shared 80% to KC937029. The aa sequence of the Pro-Pol region is 97% identical to KC937022 while the CP region is 89.8% to KC937029. A new strain of carrot torradovirus 1 (CaTV1) with similarity to CaTV1 strain celery from Germany (MK063924 and MK063925) with 95.9% aa identity to the Pro-Pol region and 95.4% to the CP region.

Finally, a sequence with 81.3% nt identity to Wuhan insect virus 21 (WHIV21; KX883227) was detected in sL of Salzlandkreis-1 2018.

### Spatial and Temporal Differences in the Pea Viruses

The spatial and temporal compositions of the virome, in the different regions over a period of three growing seasons, show similarities as well as fundamental differences. In pea crops, PEMV1, PEMV2, and PNYDV were the viruses that were found in all regions but not in every season (Figure 3). PEMV1, PEMV2 and their satellites were not detected in 2017 in Landkreis Rostock, or in 2018 in Kreis Stormarn. In 2018, PEMV1 was not detected in Landkreis Rostock, but PEMV2 and the satellite RNA were present. PNYDV was detected in all six regions in 2016 (Figure 3). In 2017, PNYDV was detected only in Salzlandkreis-1 and Landkreis Meißen. In 2018, it was detected in three regions: Salzlandkreis-1, Salzlandkreis-2, and Landkreis Meißen.

PSbMV was detected in all seasons in Salzlandkreis-2 and Kreis Stormarn. BLRV was detected only in Salzlandkreis-2 in 2016 and 2017 but it could not be found in 2018. PaEV was also detected for two seasons, in 2017 and 2018, only in Landkreis Meißen.

TuYV was detected in all three seasons in Salzlandkreis-1, Münster and Landkreis Meißen; however, after finding it present in Kreis Stormarn in 2016, but it could not be detected in 2017 and 2018. In 2017 TuYV was also not detected in Salzlandkreis-2. TuYV was never detected in the Landkreis Rostock region (Figure 3).

Other detected viruses included BYMV, detected only in Salzlandkreis-1 and Salzlandkreis-2 in 2018. ClYVV was found in Salzlandkreis-2 in 2018 and Münster in 2017. RCEV 1 and RCUV were detected only in Landkreis Meißen in 2017; however, both viruses were detected in the sL of Münster in 2016. SbDV was detected in SP in Münster 2016 and in the sL of Kreis Stormarn in 2016 and 2018. BLRV and PaEV were detected on SP only in Salzlandkreis-2 and Landkreis Meißen, respectively.

In summary, from the spatial distribution of the 12 pea viruses (excluding the mitoviruses), we observed that the highest virus occurrence was detected in Salzlandkreis-2 (17 occurrences over the three seasons) followed by Landkreis Meißen with 14 virus occurrences. A total of 13 pea virus occurrences were reported from Salzlandkreis-1, 12 for Münster, nine for Kreis Stormarn and the lowest number of occurrences was for Landkreis Rostock (only five pea viruses). The temporal virus occurrences appeared to be relatively stable over the successive seasons with the highest being 2016 with 27 occurrences, then 22 for 2017 and 21 for 2018 (Figure 3).

## Discussion

This is the first HTS-based study to describe the pea virome in Germany and to our knowledge, elsewhere in the world. In addition to the focus on viruses infecting pea crops, we also explored spatio-temporal aspects of virus infection across six different production regions over a period of 3 years. We also explored potential virus reservoirs by investigating legume and non-legume hosts in and around the crops. Our study is distinct from many metagenomics studies in that we explored our subject over three seasons in six regions. Other studies have focused on either just one crop plant, one production area, one season or a combination thereof. We believe that this study demonstrates the importance of incorporating spatio-temporal elements into metagenomics studies to help us to draw a more complete picture of all the viruses present and their impacts on the host crop as we would have missed many virus detections if we had focused on either just one growing season or just one region (e.g., the novel emaravirus that was not detected in 2016 nor outside Meißen).

To explore the pea virome dispassionately, we used ribosomal RNA-depleted total RNA for our sequencing approach (Pecman et al., 2017). This method has demonstrated it can successfully detect both RNA viruses with a plus and negative sense genome, as well as those with a DNA genome. Owing to the recent outbreaks of PNYDV in Germany and other European countries (Grigoras et al., 2010a; Gaafar et al., 2016, 2017, 2018), we were particularly interested to detect this DNA virus and its associated satellites, not only in pea crops but in particular in alternative hosts but unfortunately we were unable to identify an alternative natural host for PNYDV. We discovered a surprisingly high number of viruses in the different sample pools in high abundance, in particular, positive single-stranded viruses, including PEMV1, PEMV2, PEMVSatRNA, TuYV, TuYVaRNA1, and TuYVaRNA2. The recovered viral reads appeared to be pool-dependent as well as dependent on the viral genome, virus titer and frequency of the virus within the pool (Supplementary Tables S3, S5). It is interesting to note that also dsRNA viruses, i.e., partitivirids, and the new negative sense RNA emaravirus were detected using the ribo-depletion method, although with a lower read abundance.

### Pea Virome

#### Detection of Well Recognized Pea Viruses in Germany

Using RT-PCR, we were able to confirm the presence of pea viruses that are commonly known to be present in Germany including PEMV1, PEMV2, TuYV, PNYDV, BLRV, SbDV, PSbMV, and CMV. PEMV1 and PEMV2 are the most common viruses found in German pea crops (Ziebell, 2017). PEMV disease is associated with mosaic and enation of leaves along with pod distortion; however, early stages of infection may remain symptomless. These symptoms lead to severe yield losses (Clement, 2006). Mixed infections of PEMV1 and PEMV2 are well documented and were also common in our pea samples (Supplementary Table S4; Hagedorn and Khan, 1984; Brault et al., 2010). Interestingly, we also found PEMV2 in the absence of its helper virus PEMV1 (Landkreis Meißen 2018) and it would be interesting to know if a different helper virus would be able to facilitate PEMV2 transmission. Resistance to PEMV has been an important goal of green pea breeding for many years (Budke and Weil, pers. comm.), with many commercial varieties becoming available in the future.

The second most prevalent virus from our survey was TuYV. It was detected in pea crops and in snL pools. TuYV infects peas and may cause plant stunting and a top yellowing leaf symptom in susceptible cultivars. TuYV is also a major concern in German rapeseed crops (Graichen and Schliephake, 1999; Gaafar and Ziebell, 2019b). TuYV has a very wide host range including weeds, legume pastures and other members of the *Brassicaceae* (Stevens et al., 1994). Although we have no direct evidence that surrounding non-legumes are reservoirs for TuYV isolates that infect peas, in greenhouse experiments we demonstrated that TuYV isolates originating from peas can infect rapeseed and *vice versa* (data not shown). It is therefore very likely that rapeseed crops, other members of the *Brassicaceae* family as well as a large number of common weeds and wild species are alternative hosts for pea-infecting poleroviruses such as TuYV (Stevens et al., 1994). Resistance to luteovirids in peas is well documented. In New Zealand, it is in response to the top yellows disease complex caused by TuMV and/or SbDV, and in North America, in response to bean leafroll virus (BLRV) (Fletcher, pers. comm.). In Germany, sources of resistance have been identified in rapeseed and efforts are underway to breed TuYV-resistant rapeseed varieties (Graichen, 1994). Similarly, BRLV-resistance has been introduced into pea germplasm but it is currently not known whether it also provides resistance to TuYV. However, since BLRV was not very abundant in our survey (only found in region 2 in 2016 and 2017 in symptomatic peas), it does not seem to play a major role in the German pea virome.

In 2009, PNYDV was first described in Germany (Grigoras et al., 2010a). In the following years, PNYDV was detected in Saxony and Saxony-Anhalt, as well as in neighboring Austria (survey data, not shown). In 2016 a country-wide outbreak occurred in Germany, with PNYDV being detected in other European countries including Denmark and The Netherlands (Gaafar et al., 2016, 2017, 2018; Ziebell, 2017). Effects on infected plants are severe and can cause high yield losses (Saucke et al., 2019). PNYDV causes severe yellowing and dwarfing of infected plants that can lead eventually to plant death (Gaafar et al., 2017). PNYDV is an increasing threat to legume production in Europe as no PNYDV-resistant plant varieties have yet been identified (Ziebell, 2019). Also of concern are the high mutation rate, reassortment and recombination rates of nanoviruses such PNYDV, and these might lead to the emergence of novel strains (Grigoras et al., 2010b, 2014).

In recent years, an increasing number of nanovirus-associated single-stranded circular DNA alphasatellites have been reported in legumes, including *Sophora alopecuroides* L., *Vicia cracca* L., and *Apiaceae* members such as *Petroselinum crispum* (Mill.) Fuss. At this stage, their biological relevance is still unclear (Heydarnejad et al., 2017; Gallet et al., 2018; Vetten et al., 2019). Our survey also detected DNA alphasatellites in German peas. However, we failed to identify alternative host plants for PNYDV or its associated alphasatellites in our study. We believe by focusing on alternative plants within the pea fields or in close proximity, we may have missed potential hosts, and we need to widen the sampling radius in future surveys.

The seed-borne and aphid-transmissible potyvirus PSbMV has been reported from many countries, including Germany (Khetarpal and Maury, 1987; Latham and Jones, 2001). As the symptoms of PSbMV are often mild and transitory in peas, there is limited detection of the virus in the field. However, significant seed damage may occur in some susceptible varieties (Khetarpal and Maury, 1987). In Germany, PSbMV is not seen as a major constraint of pea production, as the provision of “clean” seed material and close surveillance of pea seed production has helped to reduce PSbMV in commercial crops to acceptable rates. One of the two sites in which we detected PSbMV is a trial site for heritage material where eradication of seed-borne virus is difficult to manage without compromising the collection. The second detection of PSbMV was found close to a protein pea breeding site where PSbMV had been previously reported (data not shown).

#### New Players in German Peas

Our study shows that in Germany, there are viruses present that have not been described from peas in Germany before: BCMV, BYMV, ClYVV, RCEV1, RCUV, and associated nucleic acids, i.e., PEMVSatRNA, TuYVaRNA, PNYDVαSat1, and 3. BCMV is well known to infect *Phaseolus* beans causing common mosaic or black root disease depending on the host, virus strain and the environmental conditions (Drijfhout and Bos, 1977). BCMV is a seed-borne virus, aphid transmissible and can be transmitted mechanically. It is interesting to find strain NL1 in German peas for the first time as previous studies showed that this strain could not infect peas (Drijfhout and Bos, 1977).

BYMV has a wide host range compare to other potyviruses including legumes and ornamentals (Guyatt et al., 1996; Nakazono-Nagaoka et al., 2009). Additionally, it can be transmitted by more than 20 aphid species causing symptoms including mosaic, necrosis and yellowing resulting in severe yield losses (Guyatt et al., 1996; Nakazono-Nagaoka et al., 2009). The pathogenicity and serotypes of the BYMV differs from a strain to another (Bos, 1970; Barnett, 1987). Clover yellow vein virus (ClYVV) has a host range overlapping with BYMV and often confused with it as they are closed serologically (Barnett, 1987; Nakazono-Nagaoka et al., 2009). Both viruses were reported in German legumes such as clover before. However, to our knowledge this is the first report of their natural infection to peas in German fields.

Interestingly, the red clover viruses RCEV1 and RCUV were not only detected in peas but also in the sL (but not in the same location and not in the same season), which indicates that more information is required to determine if surrounding perennial legumes are a virus reservoir for these viruses. Mixed infections of both viruses were confirmed in red clover (*Trifolium pratense* L.) in the Czech Republic (Koloniuk et al., pers. comm.). Relationships between luteovirids and umbraviruses are important, for example, in the relationship between PEMV1 and PEMV2 where one virus provides cell-to-cell movement within hosts and the other virus’ coat protein is required for genome packaging and vector transmission (Syller, 2003).

For the first time in Germany, we discovered numerous virus-associated nucleic acid sequences. PEMVSatRNA is a small linear single stranded RNA satellite that has also been extracted from peas in the United States (Demler and de Zoeten, 1989). PEMVSatRNA does not appear to influence aphid transmission, particle morphology, or symptom expression in peas but it can reduce the severity of symptoms in *Nicotiana benthamiana* (Demler and de Zoeten, 1989; Demler et al., 1994). Whether the PEMVSatRNA detected in Germany can modulate symptom expression of PEMV in its natural host, *P. sativum*, remains to be investigated.

We also discovered PNYDV-associated alphasatellites 1 and 3 in our survey for the first time in Germany. PNYDVαSat1 was previously detected in Austria, while PNYDVαSat3 was detected in both Austria and Denmark (Gaafar et al., 2018). In addition, we discovered four new PNYDV-associated alphasatellites that have not been reported before. Alphasatellites rely on their helper virus for spread as they do not encode a coat protein (Briddon et al., 2018). The presence of alphasatellites is associated with reduced infectivity of faba bean necrotic yellows virus (a nanovirus) and in the case of begomoviruses, alphasatellites reduced or intensified symptoms and/or reduced titre of the begomovirus or its associated betasatellites (Timchenko et al., 2006; Kon et al., 2009; Paprotka et al., 2010; Idris et al., 2011; Mar et al., 2017). The Rep proteins encoded by alphasatellites, associated with begomoviruses, were found to suppress transcriptional gene silencing or post-transcriptional gene silencing (Nawaz-Ul-Rehman et al., 2010; Abbas et al., 2019). The role of these nanovirus-associated alphasatellites is unknown and their association with nanoviruses and other viruses such as babuviruses and begomoviruses requires clarification.

Finally, TuYVaRNA and TuYVaRNA2 were also detected in association with TuYV in pea hosts. We recently discovered a TuYVaRNA that is associated with TuYV isolates from rapeseed in Germany (Gaafar and Ziebell, 2019b). Similarly, their role in symptom modulation, host range determination or vector transmission also remains to be investigated. The effects of these associated RNAs on TuYV transmission and infection need more investigation, as previous studies showed that beet western yellows associated RNA (strain ST9) increases the severity of beet western yellows on its host (Sanger et al., 1994). Interestingly, PEMVSatRNA was almost always detected in the same pools as either helper virus PEMV1 or PEMV2 (Supplementary Table S4). In contrast, the TuYVaRNAs were only detected in few pools (only in Salzlandkreis-1 and -2 as well as in Landkreis Meißen, Supplementary Table S4); although there was a high abundance of TuYV in Münster, no associated RNAs could be detected. The reasons for this observation is unknown and warrants further investigation. It would be interesting to study the transmission efficiency of aphids for the viruses in single infection or in mixed infection with associated RNAs.

#### First Report of Novel Pea Viruses

Our study in Germany identified many viruses present in peas that were not previously detected or described. PaEV, as an example, was first detected in peas over two successive seasons in Landkreis Meißen. We believe that this virus may be established and might pose a risk to pea production. We were unable to attribute clear symptoms to PaEV as when we back-tested individual plant samples from the mixed pools, PaEV was only found in mixed infection with PEMV2 and PNYDV (Supplementary Figure S5) and we could not recover infectious virus material from the samples to inoculated indicator plants. However, since we detected PaEV in SP pools, using our specific primers (Supplementary Table S2), we can specifically test peas in future surveys. Emaraviruses have mainly been reported from trees and deciduous shrubs. In the United States and Canada, the emaravirus RRV is mite-transmitted by *Phyllocoptes fructiphilus* Kiefer (Acari: Eriophyidae). RRV causes extreme damage to roses, leading to plant death within a short period of time (Olson et al., 2017). RRV and its vector were placed on the A1 alert list by the European and Mediterranean Plant Protection Organisation (EPPO, 2019). Two other emaraviruses have been reported from *Cajanus cajan* L. (*Fabaceae*): pigeonpea sterility mosaic disease (SMD), caused by pigeonpea sterility mosaic emaravirus 1, and pigeonpea sterility mosaic emaravirus 2 (Elbeaino et al., 2014, 2015). These emaraviruses are also transmitted by eriophyid mites (*Aceria cajani* Chann.) (Elbeaino et al., 2015; Patil and Kumar, 2015). One can assume that PaEV might also be a mite-transmitted virus as there are various mite species reported on pea (Annells and Ridsdill-Smith, 1994; Morishita and Takafuji, 1999; Takafuji and Morishita, 2001; Abdallah et al., 2014). The number of segments of our PaEV isolate are unknown as the virus full genome could not be recovered due to its low number of reads from the pools. In future monitoring programs, the isolation, distribution and its potential vector(s) will be evaluated to assess the risk that this virus might pose.

#### Pea-Associated Mitoviruses

Three new mitoviruses were found in the pea pools: PaMV1, PaMV2, and PaMV3. Mitoviruses are widespread in plants and their infection of pathogenic fungi is often associated with reduced virulence (Wu et al., 2007; Xie and Ghabrial, 2012; Bruenn et al., 2015). The fungi associated with these new mitoviruses are unknown and need more investigation. It is possible, for example, that these mitoviruses may have a role as biocontrol agents of fungal infections. For example, they may reduce the impact of powdery mildew, downy mildew, *Aphanomyces*, and/or fusarium root rot diseases in peas (Boland, 2004). The mitovirus SsMV4 infects *Sclerotinia sclerotiorum* (Lib.) de Bary, a widespread plant pathogenic fungus, which causes white mold disease especially in peas, lentils and beans and many other hosts (McKenzie and Morrall, 1975; Purdy, 1979; Bardin and Huang, 2001; Bolton et al., 2006; Nibert et al., 2018). Research has shown that SsMV4 in combination with two other mitoviruses, *Sclerotinia sclerotiorum* mitovirus 2 (SsMV2) and *Sclerotinia sclerotiorum* mitovirus 3 (SsMV3), reduced the *in vitro* growth and virulence of *S. sclerotiorum* on cabbage, common bean, oilseed rape and tomato (Khalifa and Pearson, 2013).

### Viruses in the Surrounding Plants

In our study, we analyzed leguminous and non-leguminous plants in the vicinity of the pea fields to investigate potential virus reservoirs. Not surprisingly, in the pools of sL we were able to detect common legume-infecting viruses, i.e., BCMV, SMV, WCCV2, WClMV, CMV, and SbDV. We also identified new viruses in the sL pool including RCCV1, RCNVA, WClMoV, WHIV21, RCEV1, and RCUV (discussed above) and a novel ilarvirus, sLaIV. In 2016, a survey using antibodies developed for detection of red clover vein mosaic virus (RCVMV)-like carlaviruses suggested the presence of a carlavirus in several pea samples but the exact virus species was not determined (Ziebell, 2017). Koloniuk et al. (pers. comm.) recently identified the genome of RCCV1 and found that its capsid protein sequence is closely related to its homolog from RCVMV but the RdRp sequence differs significantly. RCNVA is a new virus that was identified in red clover (*Trifolium pratense* L.) in the Czech Republic (Koloniuk et al., 2018). It was detected only once in 2018 in the sL pool of Landkreis Rostock. The host range of RCNVA is currently unknown. Although the exact host of new ilarvirus sLaIV is currently unknown, this virus will be included in future surveys to investigate the abundance and potential host plants. To our knowledge, sLaIV is only the second ilarvirus to naturally infect legumes apart from tobacco streak virus (Kaiser, 1982).

In the pools of non-legume (snL) plants, we detected viruses previously described in Germany. These are CaTV1, CLRV, and TuYV. TuYV was often detected and poses a threat to other commercial crop plants including rapeseed and sugar beet. CaTV1 was recently detected in celery plants, exhibiting chlorotic ringspots, mosaic and strong yellowing symptoms (Gaafar and Ziebell, 2019a). Viruses in the snL pool that have not previously been reported from Germany include BVG, BGCV2, HLV, and TVCV. We also detected several new viruses in these pools, i.e., snLaCV, snLaIV, snLaSV, and snLaWV.

In summary, our study represents the first comprehensive virome study of the pea crop covering pea production regions, temporal effects and alternative hosts. We believe that our strategy using HTS was successful. We not only detected well established pea viruses such as PEMV and PNYDV, but also discovered viruses not previously reported from Germany, as well as a number of new viruses. Furthermore, we also detected viruses in samples that appeared asymptomatic indicating that HTS is able to detect early stage or low titer infections and/or asymptomatic infections. The sequence data that we generated will also improve our knowledge of virus taxonomy, ecology, epidemiology and host plant resistance. The primer that we developed can be applied by diagnosticiancs to check for the presence of the novel viruses and virus-associated nucleic acids. It also demonstrates the challenges of metagenomic HTS studies in the framework of laboratory and bioinformatics, result interpretation, biological significance, pest risk analyses and data sharing as we also found viral reads that could not be attributed to “real” virus findings (Supplementary Table S6; Olmos et al., 2018).

While we acknowledge the strength of HTS in identifying known and unknown viruses of crops, our pooling strategy has disadvantages. Firstly, we cannot obtain detailed information on the viruses infecting a single plant without back-testing each specimen in the pool. Secondly, it was not always possible to recover the full-length viral sequence using this method. Thirdly, pooling does not allow us to link individual plants symptoms with the viruses detected.

## Conclusion

In conclusion, our method of using rRNA-depleted total RNA extracts from pooled plant tissue in combination with HTS, bioinformatic analysis and molecular confirmation has increased the speed and breadth of virus detection in one crop species in Germany, over three seasons. This method enabled the detection of a range of viruses regardless of their genome type. After sequencing our samples, we identified thirty-five viruses, for which we obtained many nearly full genomes (Supplementary Table S5). As expected, well-recognized pea viruses were detected in this study, including members of the *Luteoviridae*, *Nanoviridae*, *Potyviridae*, and *Tombusviridae* families. In addition, 25 new viruses associated with pea, non-crop legumes and non-legume plants were found, some unexpected and yet unexplained. More work is needed to reveal the importance and context of these new host/virus associations.

We found PEMV1 and PEMV2 were the dominant virus species in pea, which is consistent with what has been observed in the past. The most abundant virus was TuYV, as its sequences were not only recovered from pea pools but particularly from non-leguminous alternative host plants, which is consistent with its wide host range. A new emaravirus was detected in peas over two of the survey seasons, but its method of transmission and impact are still unknown and need further investigations. Other viruses were also detected in pea or alternative plants for the first time in Germany and their impacts have yet to be determined. Interestingly, most of the viruses detected in this survey were aphid-transmitted, thus emphasizing the continued importance of aphid management to reduce virus-spread. We also found viruses with little similarity with known species and suggest they could be categorized as unclassified. Many of these viruses have yet to be included in standard monitoring programs of pea diseases and therefore the abundance and impact of these viruses on pea and other legume crops are unknown.

We believe the data from this study provides a comprehensive and improved overview of viruses present in German pea fields, and demonstrates the usefulness of HTS and metagenomics. For the newly detected viruses, further work is needed to determine the complete host range of these viruses, their effect on hosts and their likely vectors. It is also necessary to further investigate different locations and environments to increase our understanding of the diversity of these new viruses, not only for pea crops but also for other legumes, on a global scale. Our study shows that there are many more viral players in the field(s) than known from the past. Further studies are required to address the risks that these new viruses might pose to peas or other crops. Targeted surveys could assess the distribution and abundance of these new players and the risk they might pose to crop plants.

## Data Availability Statement

The datasets presented in this study can be found in online repositories. The names of the repository/repositories and accession number(s) can be found below: https://www.ncbi.nlm.nih.gov/genbank/, MN314973, MN399680–MN399748, MN412725–MN412751, and MN497793–MN497846.

## Author Contributions

YG, JF, AB, RM, and HZ conceived and designed the experiments. YG, JF, AB, and HZ performed the sampling. YG performed the experiments, analyzed the data, and wrote the draft of the manuscript. KH and JH provided technical assistance. RM and HZ acquired the funding. All authors read and approved the final version.

## Conflict of Interest

JF, AB, and RM were employed by The New Zealand Institute for Plant and Food Research Limited, Auckland, New Zealand. The remaining authors declare that the research was conducted in the absence of any commercial or financial relationships that could be construed as a potential conflict of interest.

## Supplementary Material

Supplementary Figure 1 Neighbour joining tree (NJ) of virus isolates from *Luteoviridae* family detected in German pea fields over three successive sampling seasons in 2016, 2017 and 2018.

Supplementary Figure 2 Neighbour joining tree (NJ) of virus isolates from *Tombusviridae* family detected in German pea fields over three successive sampling seasons in 2016, 2017 and 2018.

Supplementary Figure 3 Neighbour joining trees (NJ) of PNYDV alphasatellites (PNYDVαSat) detected in German pea fields over three successive sampling seasons in 2016, 2017 and 2018 and representative alphasatellites species.

Supplementary Figure 4 Neighbour joining tree (NJ) of virus isolates from *Potyviridae* family detected in German pea fields over three successive sampling seasons in 2016, 2017 and 2018.

Supplementary Figure 5 Photo of the chlorosis symptom observed on sample R6-18-05 from Landkreis Meißen in 2018.

Supplementary Figure 6 Neighbour joining tree (NJ) of virus isolates from *Bromoviridae* family detected in German pea fields over three successive sampling seasons in 2016, 2017 and 2018.

Supplementary Figure 7 Neighbour joining tree (NJ) of virus isolates from *Secoviridae* family detected in German pea fields over three successive sampling seasons in 2016, 2017 and 2018.

Supplementary Figure 8 Neighbour joining tree (NJ) of virus isolates *Betaflexiviridae* family detected in German pea fields over three successive sampling seasons in 2016, 2017 and 2018.

Supplementary Table 1 List of primers used to confirm each virus detected by HTS samples from six German pea-growing regions (Salzlandkreis-1 [1], Salzlandkreis-2 [2], Münster [3], Kreis Stormarn [4], Landkreis Rostock [5], and Landkreis Meißen [6]) sampled over three seasons, 2016, 2017, and 2018.

Supplementary Table 2 Statistics of the raw data of the generated reads from each pool of samples from six German pea-growing regions (Salzlandkreis-1 [1], Salzlandkreis-2 [2], Münster [3], Kreis Stormarn [4], Landkreis Rostock [5], and Landkreis Meißen [6]) sampled over three seasons, 2016, 2017, and 2018.

Supplementary Table 3 List of the viruses and their associated nucleic acid satellites detected by HTS and confirmed by RT-PCR in each pool of samples from six German pea-growing regions (Salzlandkreis-1 [1], Salzlandkreis-2 [2], Münster [3], Kreis Stormarn [4], Landkreis Rostock [5], and Landkreis Meißen [6]) sampled over three seasons, 2016, 2017, and 2018. The calculated incidence of each virus are indicated.

Supplementary Table 4 List of the detected virus sequences and their associated nucleic acid satellites from six German pea-growing regions (Salzlandkreis-1 [1], Salzlandkreis-2 [2], Münster [3], Kreis Stormarn [4], Landkreis Rostock [5], and Landkreis Meißen [6]) sampled over three seasons, 2016, 2017, and 2018. Their GenBank accession numbers, their closest sequence matchess on NCBI (BLASTn), percent of coverage of the reference genomes, the number of reads and the number and size range of the contigs generated by *de novo* assembly are indicated.

Supplementary Table 5 List of the unconfirmed virus sequences detected in the pools. Their closest sequence matches on NCBI (BLASTn), and the number and size range of the contigs generated by *de novo* assembly are indicated.

Supplementary Table 6 The metadata records (sample, season, category, plant, symptoms, and average temperature) of samples from six German pea-growing regions (Salzlandkreis-1 [1], Salzlandkreis-2 [2], Münster [3], Kreis Stormarn [4], Landkreis Rostock [5], and Landkreis Meißen [6]) sampled over three seasons, 2016, 2017, and 2018.
