# Supplementary material for: Investigating the Pea Virome in Germany—Old Friends and New Players in the Field(s)
Source: Front Microbiol. 2020 Nov 13;11:583242. doi: 10.3389/fmicb.2020.583242 (PMC7691430; doi:10.3389/fmicb.2020.583242)
Supplement: Supplementary Figure 1 — Neighbour joining tree (NJ) of virus isolates from Luteoviridae family detected in German pea fields over three successive sampling seasons in 2016, 2017 and 2018. [file Data_Sheet_1.docx]

Supplementary Material

## Supplementary Figures


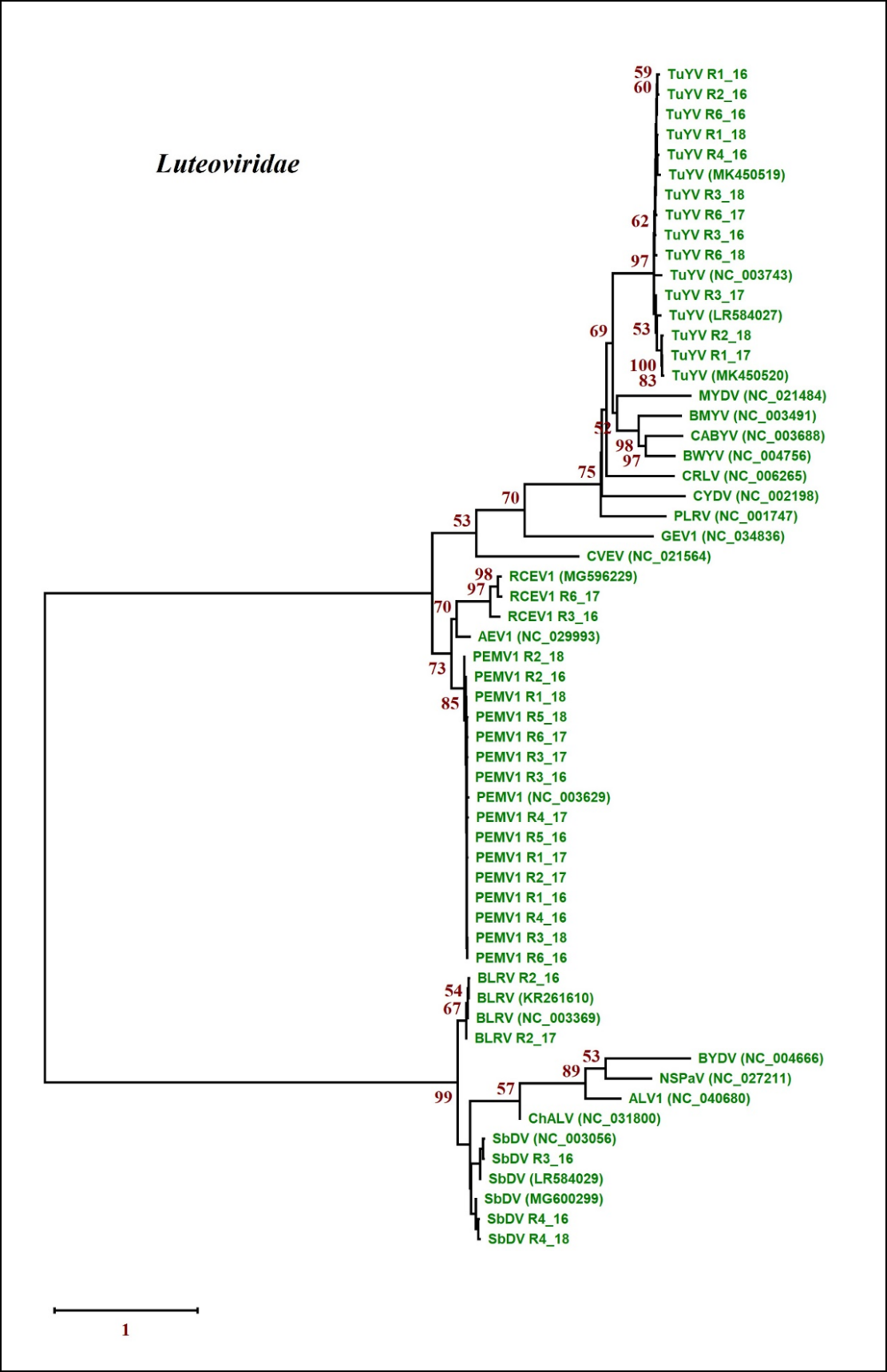


**Supplementary Figure S1.** **Neighbour joining tree (NJ) of virus isolates from *Luteoviridae* family detected in German pea fields over three successive sampling seasons in 2016, 2017 and 2018.** The phylogenetic tree is based on amino acid sequence alignments of the readthrough RNA-dependent RNA polymerases and representatives of viruses from the family *Luteoviridae*. Amino acid sequences were aligned with Clustal W and NJ trees constructed with MEGA X. The percentage of the bootstrap values above 50% (1,000 replications) are shown at the nodes. The names of the viruses are as follow: AEV1: alfalfa enamovirus 1, ALV1: apple luteovirus 1, BLRV: bean leafroll virus, BMYV: beet mild yellowing virus, BYDV: barley yellow dwarf virus, BWYV: beet western yellows virus, CABYV: cucurbit aphid-borne yellows virus, ChALV: cherry associated luteovirus, CRLV: carrot red leaf virus, CVEV: citrus vein enation virus, CYDV: cereal yellow dwarf virus, GEV1: grapevine enamovirus-1, MYDV: maize yellow dwarf virus, NSPaV: nectarine stem pitting-associated virus, PEMV1: pea enation mosaic virus 1, PLRV: potato leafroll virus, RCEV1: red clover enamovirus 1, SbDV: soybean dwarf virus and TuYV: turnip yellows virus. The scale bar indicates the genetic distance.


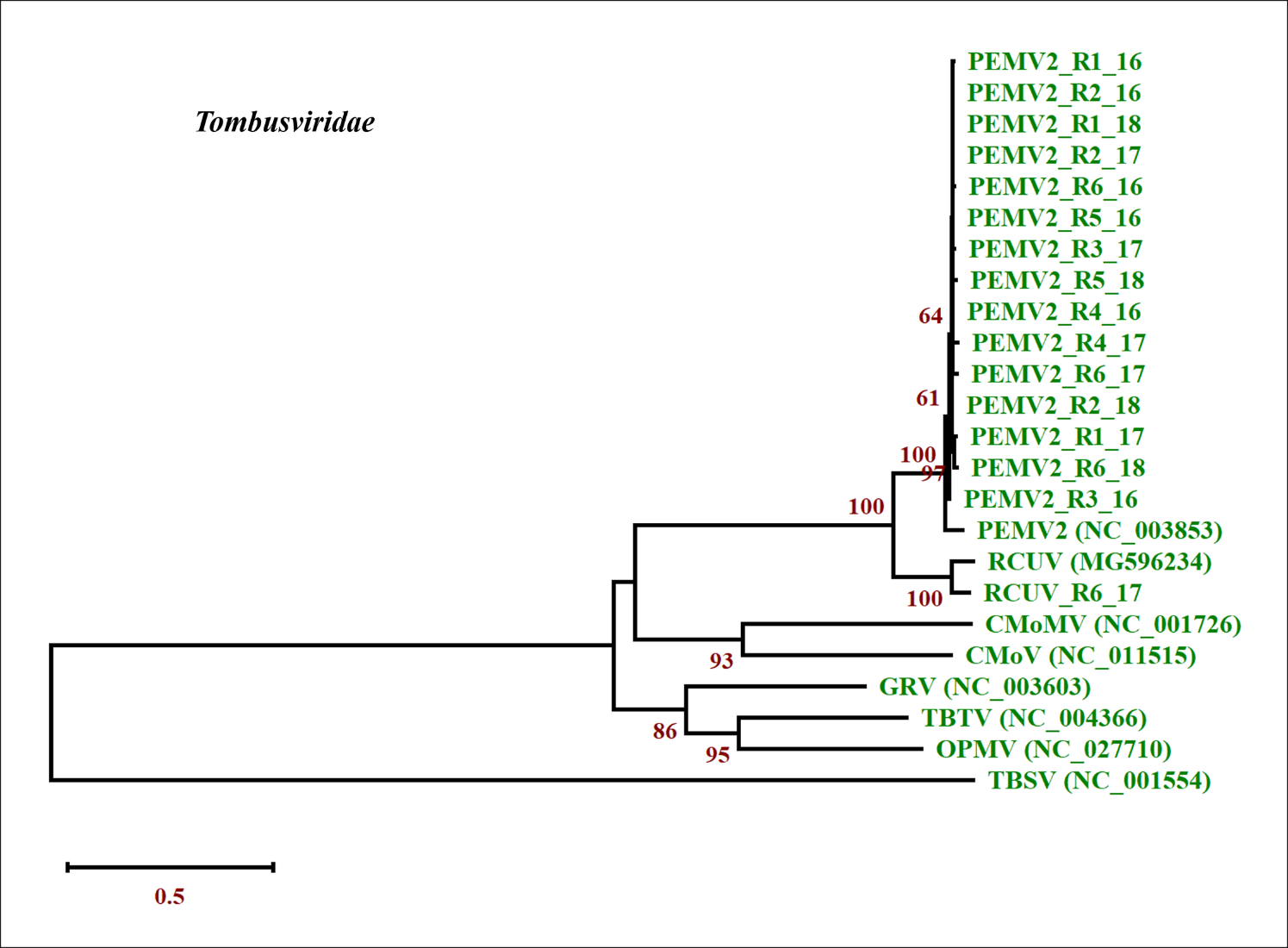


**Supplementary Figure S2.** **Neighbour joining tree (NJ) of virus isolates from *Tombusviridae* family detected in German pea fields over three successive sampling seasons in 2016, 2017 and 2018.** The phylogenetic tree is based on amino acid sequence alignments of the readthrough RNA-dependent RNA polymerases and representatives of viruses from the family *Tombusviridae*. Amino acid sequences were aligned with Clustal W and NJ trees constructed with MEGA X. The percentage of the bootstrap values above 50% (1,000 replications) are shown at the nodes. The names of the viruses are as follow: CMoMV: carrot mottle mimic virus, CMoV: carrot mottle virus, GRV: groundnut rosette virus, OPMV: Opium poppy mosaic virus, PEMV2: pea enation mosaic virus 2, RCUV: red clover umbravirus, TBTV: tobacco bushy top virus and TBSV: tomato bushy stunt virus. The scale bar indicates the genetic distance.


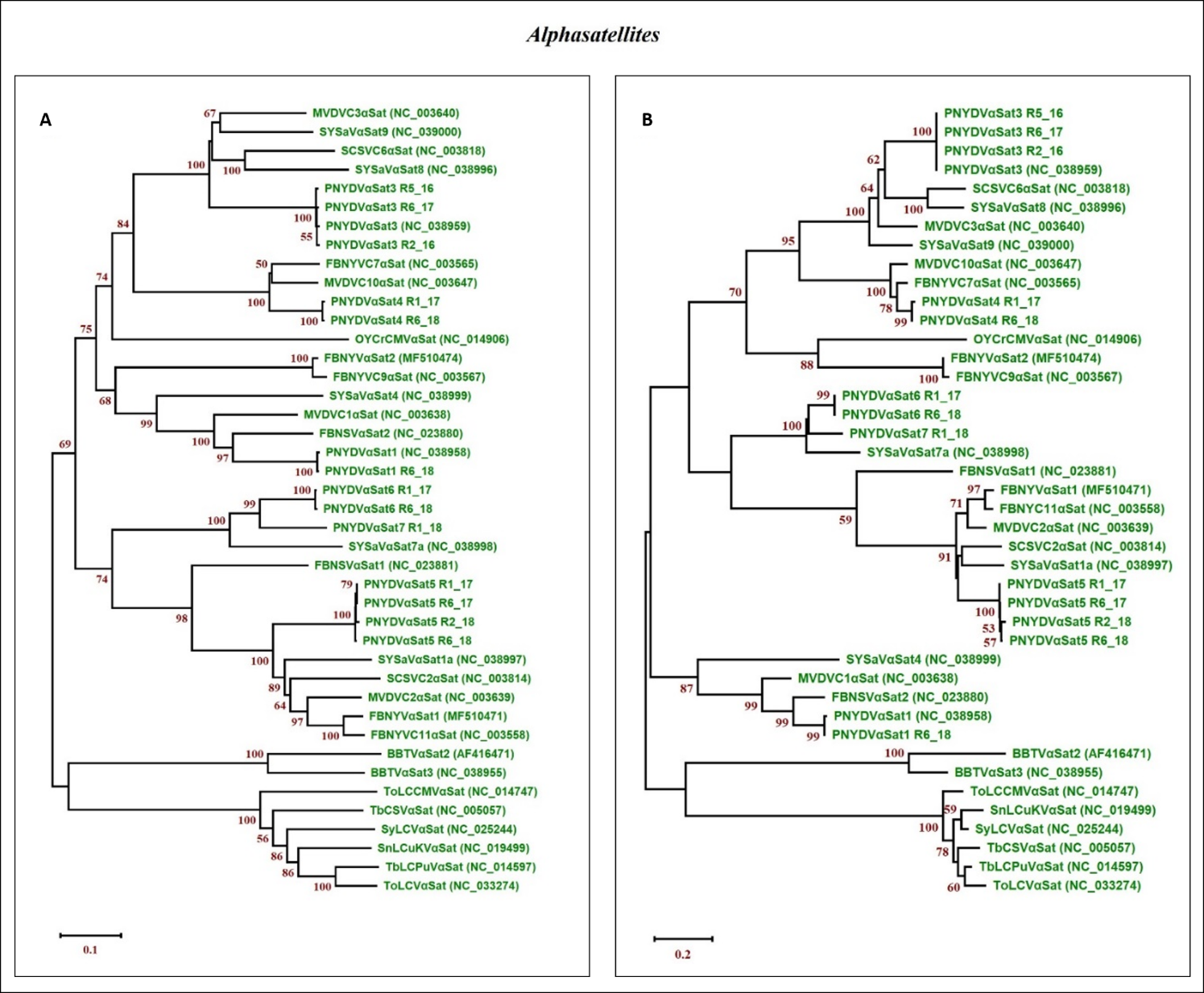


**Supplementary Figure S3. Neighbour joining trees (NJ) of PNYDV alphasatellites (PNYDVαSat) detected in German pea fields over three successive sampling seasons in 2016, 2017 and 2018 and representative alphasatellites species.** The phylogenetic trees are based on the alignments of: (A) the full-length nucleotide sequences and (B) the amino acid sequences of the coding sequences. The sequences were aligned with Clustal W and NJ trees constructed with MEGA X. The percentage of the bootstrap values above 50% (1,000 replications) are shown at the nodes. The names of the alphasatellites are as follow: BBTVαSat2: banana bunchy top virus alphasatellite 2, BBTVαSat3: banana bunchy top virus alphasatellite 3, FBNSVαSat1: faba bean necrotic stunt virus alphasatellite 1, FBNSVαSat2: faba bean necrotic stunt virus alphasatellite 2, FBNYVαSat1: faba bean necrotic yellows virus alphasatellite 1, FBNYVαSat2: faba bean necrotic yellows virus alphasatellite 2, FBNYVC7αSat: faba bean necrotic yellows virus C7 alphasatellite, FBNYVC9αSat: faba bean necrotic yellows virus C9 alphasatellite, FBNYC11αSat: faba bean necrotic yellows virus C11 alphasatellite, MVDVC1αSat: milk vetch dwarf virus C1 alphasatellite, MVDVC2αSat: milk vetch dwarf virus C2 alphasatellite, MVDVC3αSat: milk vetch dwarf virus C3 alphasatellite, MVDVC10αSat: milk vetch dwarf virus C10 alphasatellite, OYCrCMVαSat: Okra yellow crinkle Cameroon virus alphasatellite, PNYDVαSat1: pea necrotic yellow dwarf virus alphasatellite 1, PNYDVαSat3: pea necrotic yellow dwarf virus alphasatellite 3, PNYDVαSat4: pea necrotic yellow dwarf virus alphasatellite 4, PNYDVαSat5: pea necrotic yellow dwarf virus alphasatellite 5, PNYDVαSat6: pea necrotic yellow dwarf virus alphasatellite 6, PNYDVαSat7: pea necrotic yellow dwarf virus alphasatellite 7, SCSVC2αSat: subterranean clover stunt virus C2 alphasatellite, SCSVC6αSat: subterranean clover stunt virus C6 alphasatellite, SnLCuKVαSat: alphasatellite, SyLCVαSat: Synedrella leaf curl virus alphasatellite, SYSaVαSat1a: Sophora yellow stunt associated virus alphasatellite 1a, SYSaVαSat4: Sophora yellow stunt associated virus alphasatellite 4, SYSaVαSat7a: Sophora yellow stunt associated virus alphasatellite 7a, SYSaVαSat8: Sophora yellow stunt associated virus alphasatellite 8, SYSaVαSat9: Sophora yellow stunt associated virus alphasatellite 9, TbCSVαSat: tobacco curly shoot virus alphasatellite, TbLCPuVαSat: tobacco leaf curl Pusa virus alphasatellite, ToLCCMVαSat: tomato leaf curl Cameroon virus alphasatellite and ToLCVαSat: tomato leaf curl virus alphasatellite. The scale bar indicates the genetic distance.


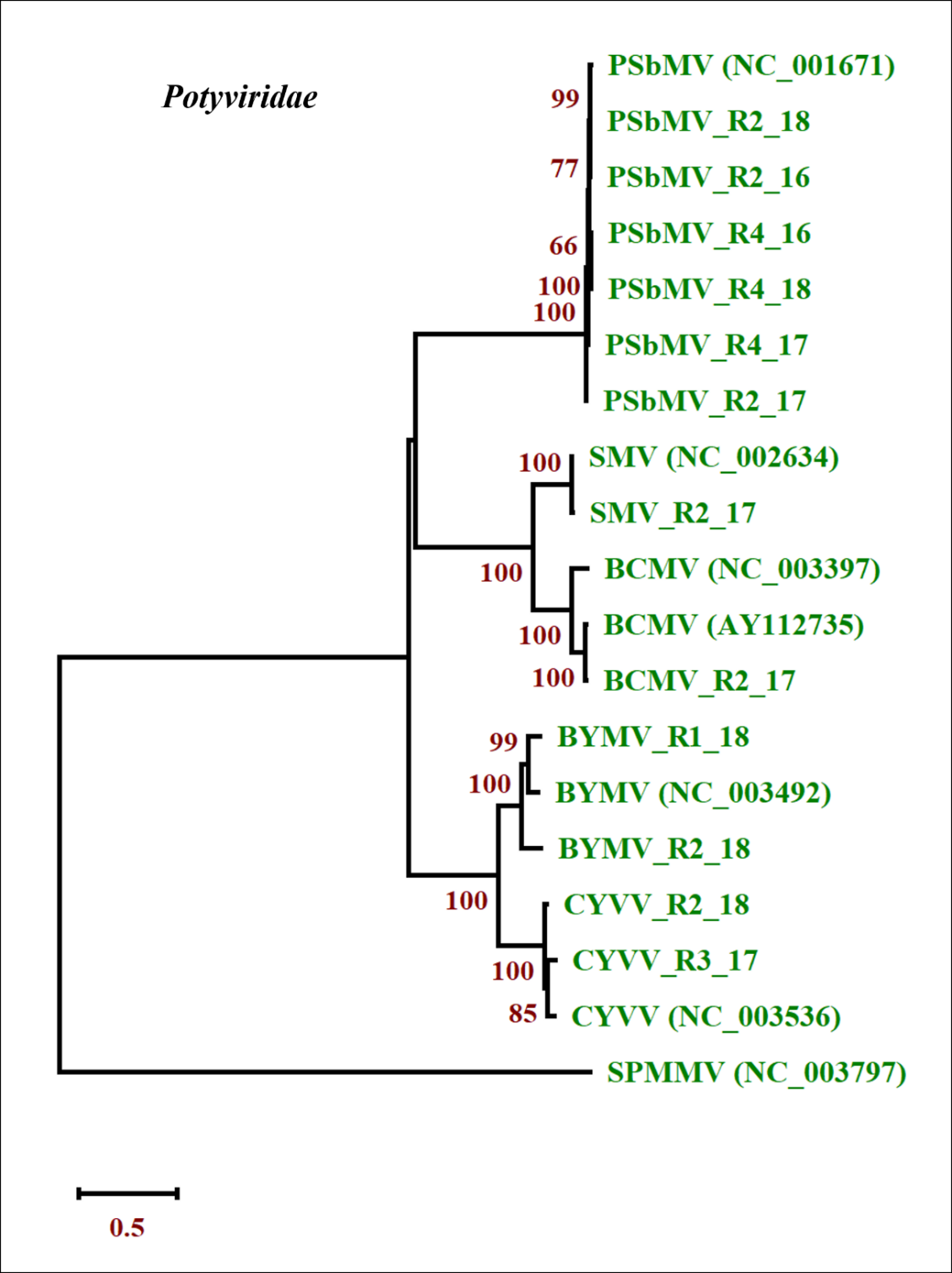


**Supplementary Figure S4.** **Neighbour joining tree (NJ) of virus isolates from *Potyviridae* family detected in German pea fields over three successive sampling seasons in 2016, 2017 and 2018.** The phylogenetic tree is based on amino acid sequence alignment of the polyproteins of the potyviruses in the survey with representative species from the family *Potyviridae*. Amino acid sequences were aligned with Clustal W and NJ trees constructed with MEGA X. The percentage of the bootstrap values above 50% (1,000 replications) are shown at the nodes. The names of the viruses are as follow: BCMV: bean common mosaic virus, BYMV: bean yellow mosaic virus, ClYVV: clover yellow vein virus, PSbMV: pea seed-borne mosaic virus, SMV: soybean mosaic virus and SPMMV: sweet potato mild mottle virus. The scale bar indicates the genetic distance.


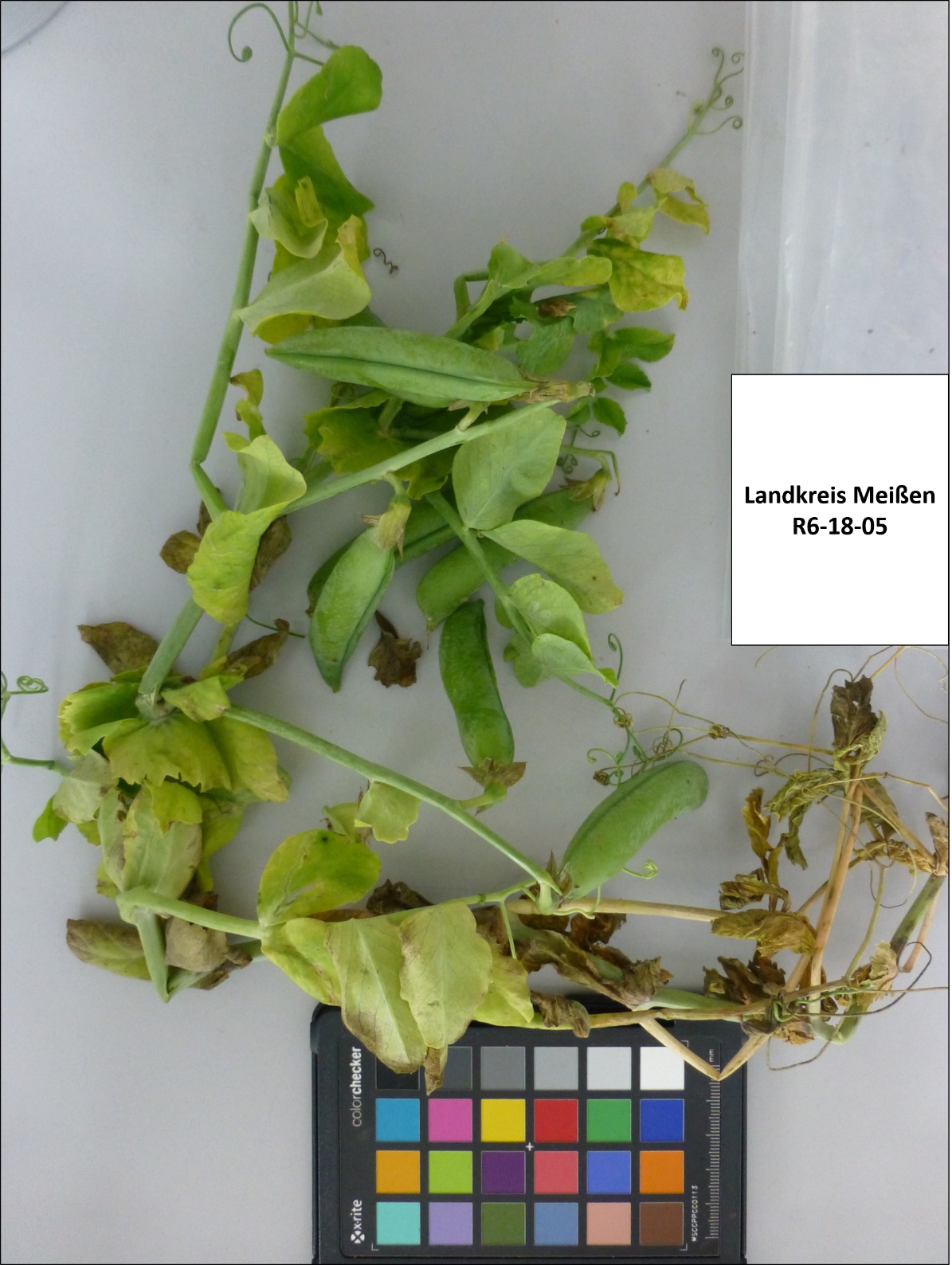


**Supplementary Figure S5. Photo of the chlorosis symptom observed on sample R6-18-05 from Landkreis Meißen in 2018.** The sample has a mixed infection with the new pea-associated emaravirus, pea enation mosaic virus 2 and pea necrotic yellow dwarf virus.


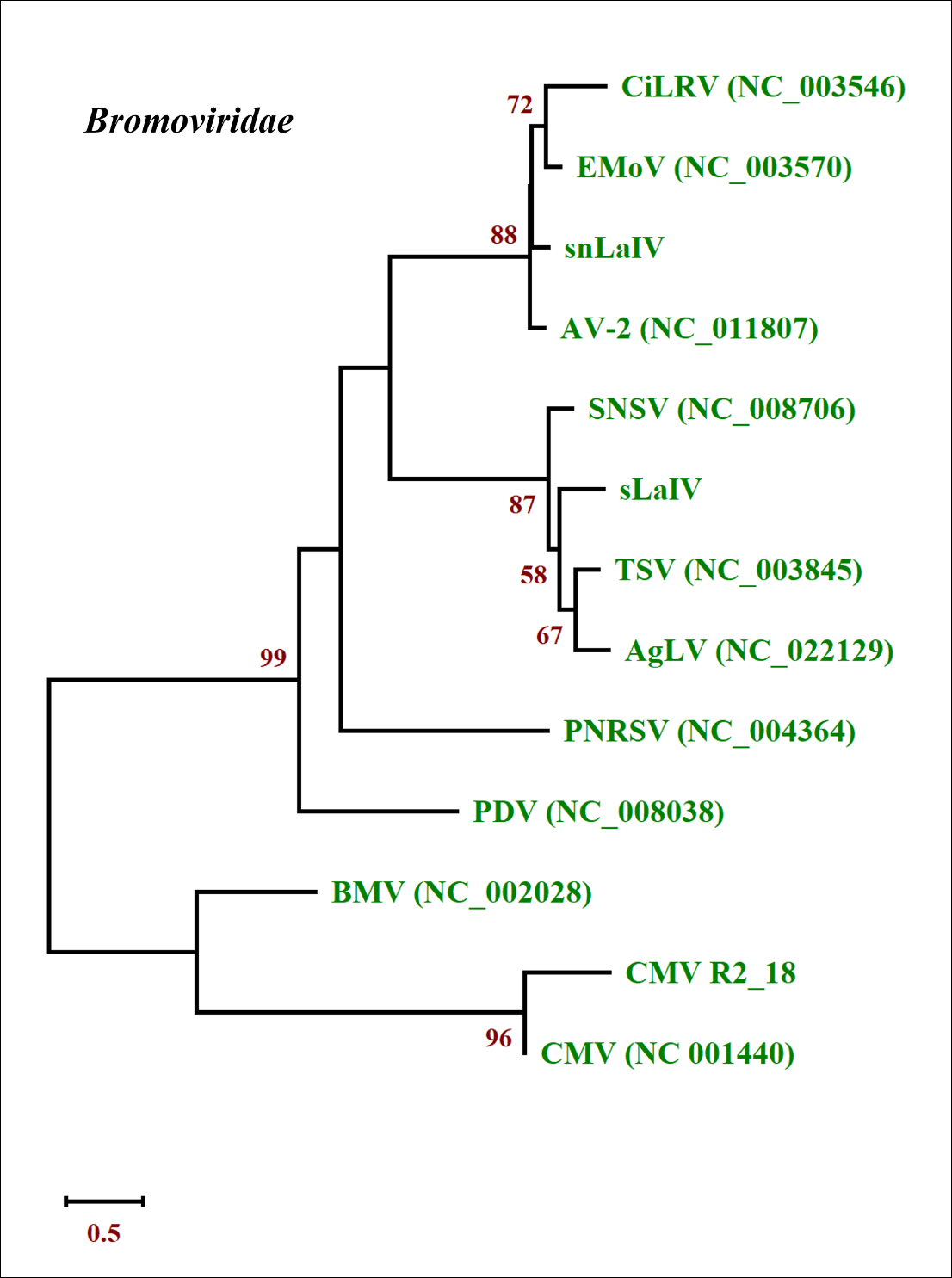


**Supplementary Figure S6. Neighbour joining tree (NJ) of virus isolates from *Bromoviridae* family detected in German pea fields over three successive sampling seasons in 2016, 2017 and 2018.** The phylogenetic tree is based on amino acid sequence alignment of the RNA-dependent RNA polymerases of bromovirids in the survey with representative species from the family *Bromoviridae*. Amino acid sequences were aligned with Clustal W and NJ trees constructed with MEGA X. The percentage of the bootstrap values above 50% (1,000 replications) are shown at the nodes. The names of the viruses are as follow: Ageratum latent virus (AgLV), Asparagus virus 2 (AV-2), Brome mosaic virus (BMV), citrus leaf rugose virus (CiLRV), cucumber mosaic virus (CMV), Elm mottle virus (EMoV), prune dwarf virus (PDV), Prunus necrotic ringspot virus (PNRSV), strawberry necrotic shock virus (SNSV), surrounding legume associated ilarvirus (sLaIV), surrounding non legume associated ilarvirus (snLaIV) and tobacco streak virus (TSV). The scale bar indicates the genetic distance.


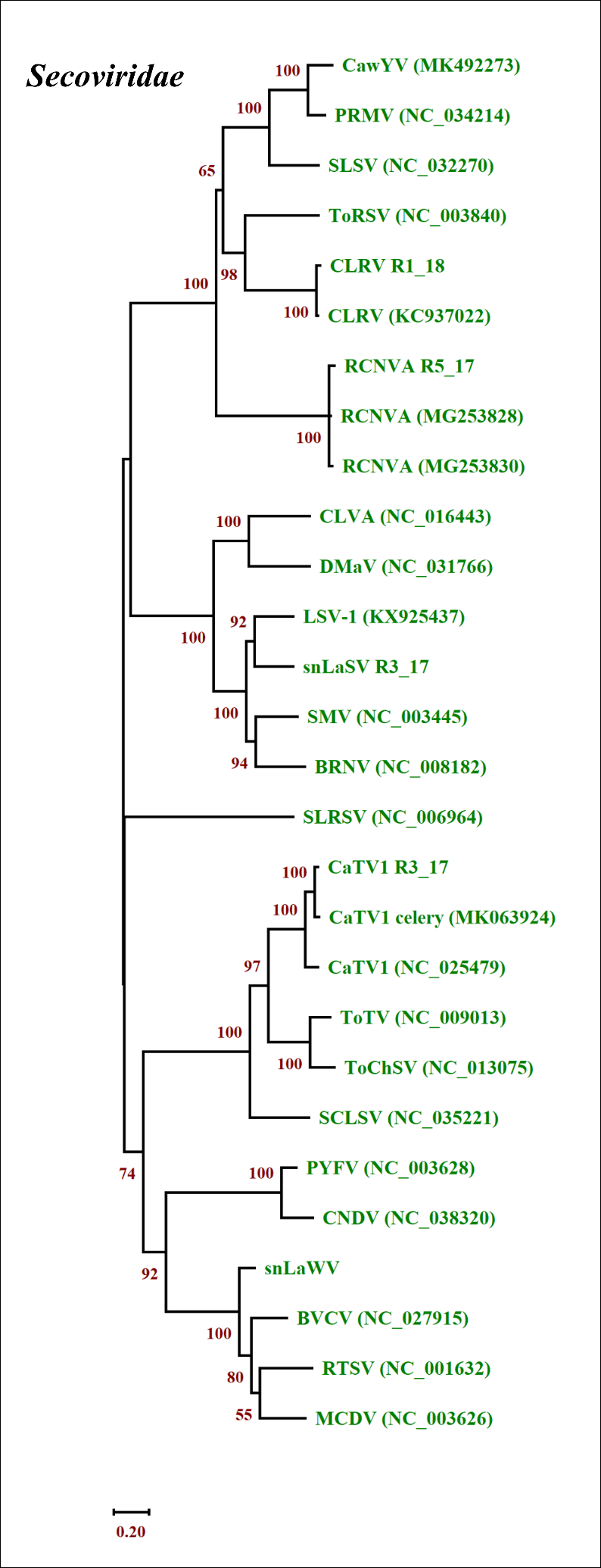


**Supplementary Figure S7. Neighbour joining tree (NJ) of virus isolates from *Secoviridae* family detected in German pea fields over three successive sampling seasons in 2016, 2017 and 2018.** The phylogenetic tree is based on amino acid sequence alignment of the protease-polymerase region of the secovirids in the survey with representative species from the family *Secoviridae*. Amino acid sequences were aligned with Clustal W and NJ trees constructed with MEGA X. The percentage of the bootstrap values above 50% (1,000 replications) are shown at the nodes. The names of the viruses are as follow: bellflower vein chlorosis virus (BVCV), black raspberry necrosis virus (BRNV), caraway yellows virus (CawYV), carrot necrotic dieback virus (CNDV), carrot torradovirus 1 (CaTV1), cherry leaf roll virus (CLRV), chocolate lily virus A (CLVA), Dioscorea mosaic-associated virus (DMaV), lettuce secovirus 1 (LSV-1), maize chlorotic dwarf virus (MCDV), parsnip yellow fleck virus (PYFV), peach rosette mosaic virus (PRMV), red clover nepovirus A (RCNVA), rice tungro spherical virus (RTSV), squash chlorotic leaf spot virus (SCLSV), soybean latent spherical virus (SLSV), strawberry latent ringspot virus (SLRSV), strawberry mottle virus (SMV), surrounding non-legume associated secovirus (snLaSV), surrounding non-legume associated waikavirus (snLaWV), tomato ringspot virus (ToRSV), tomato chocolate spot virus (ToChSV) and tomato torrado virus (ToTV). The scale bar indicates the genetic distance.


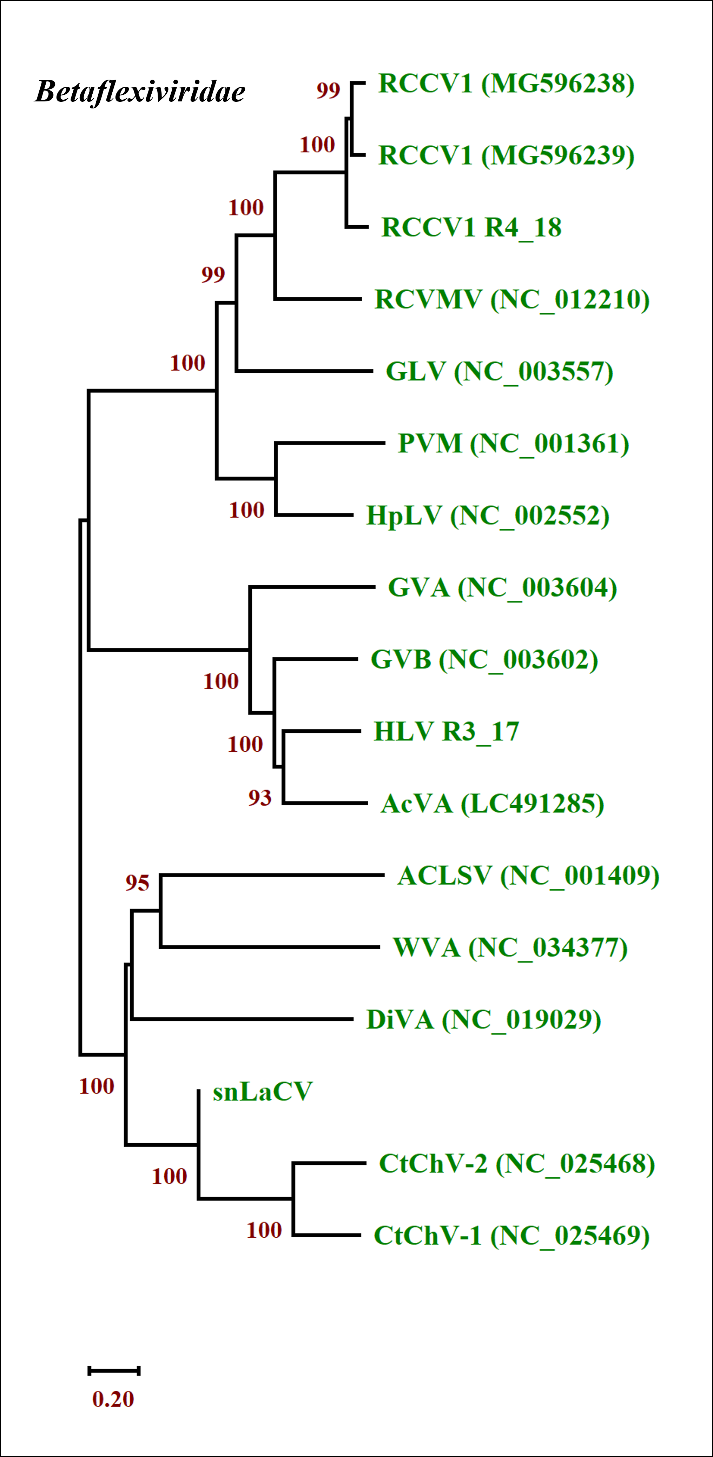


**Supplementary Figure S8. Neighbour joining tree (NJ) of virus isolates *Betaflexiviridae* family detected in German pea fields over three successive sampling seasons in 2016, 2017 and 2018.** The phylogenetic tree is based on amino acid sequence alignment of the replicase of the betaflexivirids in the survey with representative species from the family *Betaflexiviridae*. Amino acid sequences were aligned with Clustal W and NJ trees constructed with MEGA X. The percentage of the bootstrap values above 50% (1,000 replications) are shown at the nodes. The names of the viruses are as follow: Actinidia virus A (AcVA), apple chlorotic leafspot virus (ACLSV), carrot betaflexivirus 1 (CtChV-1), carrot betaflexivirus 2 (CtChV-2), Diuris virus A (DiVA), garlic latent virus (GLV), grapevine virus A (GVA), grapevine virus B (GVB), Heracleum latent virus (HLV), hop latent virus (HpLV), potato virus M (PVM), red clover carlavirus 1 (RCCV1), red clover vein mosaic virus (RCVMV), surrounding non-legume associated carlavirus (snLaCV) and watermelon virus A(WVA). The scale bar indicates the genetic distance.
